# Enhancing Neonatal Intensive Care Unit (NICU) Paternal Satisfaction: A Comparative Study of Traditional vs. Generative AI-Integrated Counseling

**DOI:** 10.7759/cureus.94374

**Published:** 2025-10-12

**Authors:** Aayushi Joshi, Shantanu Shubham, Syed Moiz Ahmed, Divya Mishra, Richa Joshi, Naini Puri, Girish Gupta

**Affiliations:** 1 Pediatrics, Graphic Era Deemed to be University, Dehradun, IND; 2 Neonatology, Graphic Era Deemed to be University, Dehradun, IND; 3 Obstetrics and Gynecology, Graphic Era Deemed to be University, Dehradun, IND

**Keywords:** artificial intelligence, chatgpt, neonatology, nicu, parental counseling, patient satisfaction

## Abstract

Background: Admission of a newborn to the neonatal intensive care unit (NICU) is a stressful experience for parents. Effective communication is critical but often constrained by time and variability in clinician delivery. Integrating AI chatbots like ChatGPT may improve parental satisfaction.

Methods: In this observational study conducted in a Level III NICU in North India, 180 fathers were quasi-randomly assigned to receive either traditional clinician-led counseling or AI-integrated counseling using ChatGPT. The primary outcome was paternal satisfaction, measured by a custom-developed 10-item NICU Parental Satisfaction Score (range: 10-50).

Results: The AI-integrated group reported significantly higher satisfaction scores (median: 41, IQR: 40-50) compared to the traditional group (median: 35, IQR: 30-45, p < 0.0001). No participant in the AI group scored <30, whereas 25% of the traditional group did. Baseline demographics and neonatal characteristics were comparable.

Conclusion: Generative AI-enhanced counseling improved parental experience in the NICU without compromising clinician interaction. With proper safeguards and clinician oversight, AI integration can enhance clarity, accessibility, and empathy in neonatal communication.

## Introduction

Admission of a newborn to the neonatal intensive care unit (NICU) is a highly stressful event for parents. They must cope with an unfamiliar clinical environment, fear for their infant's survival, and a flood of complex information [1,2]. In this scenario, effective communication and counseling by the healthcare team are not luxuries but essential needs for families [3-5]. Studies have shown that parents of NICU infants experience significant psychological distress; for example, a recent cross-sectional study in India found high levels of stress and anxiety in both mothers and fathers, with mothers being particularly affected [6]. Ensuring that parents understand their baby's condition and feel supported can help alleviate this stress and improve their coping and participation in care.

Traditionally, NICU parental counseling is delivered through face-to-face conversations with physicians and nurses, often during daily rounds or dedicated meetings. These interactions allow clinicians to explain the infant’s diagnosis, treatment plan, and prognosis, as well as to provide emotional support. Satisfying as this can be, the quality and consistency of traditional counseling are challenged by practical limitations. Even in well-resourced NICUs, research indicates that parents often desire more information and clearer communication than they receive, and they may not always feel comfortable asking all their questions [7]. Many turn to external sources such as internet forums, pamphlets, or mobile apps to fill information gaps [8-11]. This reliance on outside information can be a double-edged sword; while supplemental resources can empower parents, they also carry the risk of inaccuracies or increased anxiety if the information is not tailored to the parent's context.

In recent years, digital health innovations have been explored to enhance patient and caregiver education. Simple interventions, such as web-based information programs, have yielded positive results. For instance, a quasi-experimental study showed that providing NICU mothers with a structured internet-based education program significantly increased their satisfaction and sense of preparedness [12]. Mobile health (mHealth) applications have also been proposed to improve communication; an exploratory needs assessment in the Netherlands found that both parents and clinicians believed that a neonatal app for sharing updates, answering questions, and providing educational content could improve the NICU experience [7].

Artificial intelligence (AI), particularly generative AI-driven conversational agents (chatbots), represents the next frontier in healthcare communication [13]. AI chatbots are software programs that use natural language processing (NLP) and machine learning (ML) to engage in human-like dialogue. Trained on vast datasets of medical knowledge, they can instantly provide evidence-based answers to user queries. In HIV care, a pilot study in Singapore instructed ChatGPT to answer common questions about antiretroviral therapy; the AI provided accurate and comprehensive information and was suggested as a useful adjunct in counseling patients about treatment initiation and adherence [14]. These global developments suggest that AI could help bridge gaps in healthcare communication by delivering reliable information and guidance at patients' fingertips.

Despite this potential, the application of AI chatbots in neonatology, especially for direct parent counseling, remains nascent. Most AI efforts in NICUs to date have focused on improving clinical care through diagnostics and monitoring. For instance, ML models have been developed to predict neonatal sepsis earlier, optimize ventilator settings, or interpret neonatal brain imaging [15]. These innovations highlight AI's ability to transform neonatology through improved medical decision-making [15]. There are unique challenges in the context of counseling in the NICU. Conversations involve sensitive, emotionally charged topics (such as prognoses or end-of-life decisions), and parents' needs must be met with utmost compassion and clarity. It is in these human-centric aspects of care that skepticism about AI often arises. Critics point out that AI might lack the empathy and moral judgment required for delicate conversations, potentially leading to depersonalization of care [16]. Furthermore, concerns about information accuracy, data privacy, and the "black-box" nature of AI decisions (where even developers cannot fully explain why an AI gave a certain response) pose ethical questions [16]. Healthcare providers themselves have mixed perceptions; a qualitative study of NICU nurses in Turkey found that while many saw AI tools as valuable for saving time and simplifying information delivery, they were also worried about reduced human interaction, loss of professional autonomy, and the ethics of relying on AI in patient counseling [17].

Given these gaps and considerations, we identified a need to rigorously evaluate AI-integrated counseling within the NICU setting. Our research aimed to determine whether supplementing parental counseling with an AI chatbot (OpenAI's ChatGPT) could improve parental satisfaction without undermining the quality of the interaction.

## Materials and methods

Study design and setting

This was an interventional, cross-sectional study conducted over a six-month period in the NICU at Graphic Era Institute of Medical Sciences, Dehradun, a tertiary care teaching hospital in North India. The NICU is a Level III facility providing advanced neonatal care. Ethical approval was obtained from the Institutional Ethics Committee of Graphic Era Institute of Medical Sciences, and written informed consent was obtained from all participants prior to enrollment.

Study participants

The study specifically evaluated paternal satisfaction with NICU counseling. Fathers of neonates admitted to the NICU were eligible for inclusion if they were present within 24 hours of admission and able to participate in a counseling session. Exclusion criteria included cognitive impairment, any psychiatric illness, or substance abuse. Mothers were excluded to maintain uniformity and due to unavailability during early postnatal recovery. A total of 180 eligible fathers consented to participate and were alternately allocated (quasi-randomly by admission order) into two groups of 90 each.

Study groups

Control Group (Traditional Counseling)

Fathers in this group received the standard NICU admission counseling from a neonatologist, conducted in a designated counseling room. The session included an explanation of the neonate's clinical condition (gestation, birth weight, diagnosis, and treatment plan), a discussion of investigations and prognosis, and an opportunity to ask questions. Emphasis was placed on empathetic, clear communication, and it was scored using a checklist (Annexure 1).

Intervention Group (AI-Integrated Counseling)

In addition to the standard counseling described above, these participants underwent an AI-augmented session. Following the initial discussion, fathers were encouraged to raise further questions. A research assistant entered these queries into ChatGPT (GPT 4o, OpenAI, San Francisco, California), a large language model-based chatbot. AI-generated responses were reviewed by a clinician for accuracy and clarity, translated into the local language (Hindi) as needed, and then relayed verbally to the participant. This preserved clinician oversight while enhancing information delivery. The AI was used as a real-time reference tool, not a replacement for human interaction.

All counseling sessions were conducted in a quiet, private room within the NICU to ensure confidentiality. Sessions typically occurred on the day of admission or within 24 hours and lasted not more than 30 minutes. AI-assisted sessions occasionally took longer if more questions were addressed. Common themes of paternal queries included disease explanation, prognosis, treatment details, and potential complications, which were similar across both groups.

Outcome measures

The primary outcome was paternal satisfaction with the counseling session, measured immediately after the session using the NICU. Based on a comprehensive literature review, a 10-item structured questionnaire developed for this study (Annexure 1) was used to assess parental satisfaction in the NICU. It encompasses key domains of the counseling experience (clarity of information, parental understanding, communication, emotional support, and overall satisfaction), with each item rated on a five-point Likert scale from 'not satisfied at all' to 'completely satisfied.' Content validity was independently established by a panel of three subject experts from different academic institutions, who reviewed the questionnaire to ensure that all items were clear, relevant, and representative of the intended constructs. Items covered clarity, empathy, reassurance, opportunity to ask questions, adequacy of responses, respect for concerns, and overall satisfaction. Total scores ranged from 10 to 50. Fathers in the AI group assessed the entire counseling experience (standard + AI-augmented). Secondary data included parental demographics (age, education, and religion) and infant clinical variables (gestational age, birth weight, gender, diagnosis, and Apgar score). The timing of counseling (the same day vs. later) was also recorded.

Sample size estimation

Parental satisfaction with traditional NICU counseling has been reported at approximately 60% [18], whereas AI-supported interventions have increased satisfaction to about 80% [19]. Assuming these proportions (p₁ = 0.60, p₂ = 0.80), with a two-sided α of 0.05 and 80% power, the required sample size per group was calculated using the standard formula for comparing two independent proportions. The following formula was used for sample size calculation (n):

$n = (Z&alpha;/2+Z&beta;)2 * (p1(1-p1)+p2(1-p2)) / (p1-p2)2$

Where Zα/2 is the critical value of the normal distribution at α/2 (e.g. for a confidence level of 95%, α is 0.05 and the critical value is 1.96), Zβ is the critical value of the normal distribution at β (e.g. for a power of 80%, β is 0.2 and the critical value is 0.84), and p1 and p2 are the expected sample proportions of the two groups.

To account for attrition, 90 fathers per group were enrolled (total N = 180). All participants completed the study, and there were no missing data for the primary outcome.

Statistical analysis

Data were entered in Microsoft Excel 2016 (Microsoft Corporation, Redmond, Washington) and analyzed using STATA version 14 (StataCorp, College Station, Texas). Satisfaction scores were assessed for normality using the Shapiro-Wilk test and found to be non-normally distributed. Descriptive analysis was performed for all study variables. Continuous data were expressed as medians along with their corresponding interquartile ranges (IQR), while categorical data were described using proportions. To assess differences between the two groups, the Mann-Whitney U test was applied for non-normally distributed continuous variables, and the chi-square test was employed for analyzing differences in categorical outcomes. A p-value less than 0.05, using a two-sided test, was considered indicative of statistical significance.

## Results

Parental satisfaction was evaluated using a structured questionnaire based on 10 distinct parameters. Each parameter was scored on a scale from 1 (minimum) to 5 (maximum), resulting in a total satisfaction score ranging from 10 to 50 per participant (Annexure 1).

A total of 180 fathers were enrolled in the study: 90 in the traditional counseling group and 90 in the AI-integrated counseling group. The mean parental satisfaction score was significantly higher in the AI-integrated group (mean = 42.34 ± 3.12) compared to the traditional group (mean = 37.17 ± 2.96), with median scores of 41 vs. 35, respectively. This difference was statistically significant (Wilcoxon rank-sum test, p < 0.0001). The AI group demonstrated an IQR of 40-50 and a minimum score of 30, whereas the traditional group had an IQR of 30-45 and a minimum score of 14 (Table 1).

**Table 1:** Comparison of parental satisfaction scores between study groups SD: standard deviation; IQR: interquartile range

Statistical Measure	AI-Integrated Counseling (n=90)	Traditional Counseling (n=90)	P-value
Mean score (±SD)	42.34 (3.12)	37.17 (2.96)	0.001
Median score (IQR)	41 (40–50)	35 (30–45)	0.001
Range	30–50	14–50	-

The AI group exhibited a more favorable distribution of scores, with no participant scoring below 30, and several rating the session as perfect (50/50). In contrast, scores in the traditional group ranged as low as 14. In the traditional group, 25% of fathers scored ≤30, while 75% of those in the AI group scored ≥40. This suggests that AI integration not only increased average satisfaction but also reduced instances of low satisfaction. Among fathers of critically ill neonates (e.g., extreme prematurity or complex diagnoses), satisfaction scores were generally lower in the traditional group, likely reflecting heightened parental stress. However, in the AI group, even these subgroups showed marked improvement, with satisfaction scores approaching those of parents of stable neonates. This suggests that AI-assisted sessions may be especially beneficial in complex clinical scenarios where information needs are higher.

Baseline demographic and clinical variables were similar between the two groups (Table 2).

**Table 2:** Baseline data of the two counseling modes LGA: large for gestational age, SGA: small for gestational age, AGA: appropriate for gestational age, NNJ: neonatal jaundice, RDS: respiratory distress syndrome, LONS: late-onset neonatal sepsis, TTNB: transient tachypnea of newborn

Variable	Category	Frequency (n) Traditional Counseling	Frequency (n) AI-Assisted Counseling	P-value
Gender	Male	48	45	0.547
	Female	42	45	
Weight for Age	LGA	4	2	0.713
	SGA	5	8	
	AGA	81	80	
Gestation	< 28+6 wks	6	2	0.505
	29–36+6 wks	18	24	
	≥ 37wks	66	64	
Birthweight	<1.0 kg	7	2	0.222
	<1.5 kg	5	8	
	<2.5 kg	51	43	
	<3.5 kg	24	33	
	<4.5 kg	2	3	
	>4.5 kg	1	1	
Mode of Delivery	Vaginal	38	32	0.465
	LSCS	52	58	
APGAR at 5 mins	<7	6	3	0.289
	>7	84	87	
Day of Life of Counseling	Day 1	48	57	0.281
	Day 2–5	35	27	
	>Day 6	7	6	
Father’s Age	21–30 yrs	41	42	0.842
	31–40 yrs	43	46	
	41–50 yrs	6	2	
Religion	Hindu	54	64	0.548
	Muslim	29	22	
	Sikh	5	3	
	Others	2	1	
Education	< Secondary	12	12	0.430
	Secondary	33	35	
	Higher Secondary	21	13	
	Graduate	24	30	
Diagnosis	NNJ	30	19	0.484
	RDS	6	12	
	LONS	4	11	
	TTNB	9	5	
	Others	41	43	

The gender distribution among newborns was nearly equal (male: 51.7%; female: 48.3%), with no significant difference between groups (p = 0.547). Most neonates were appropriate for gestational age (AGA: 89.4%), followed by small for gestational age (SGA: 7.2%) and large for gestational age (LGA: 3.3%), with comparable distributions across study arms (p = 0.713). The majority of infants were born at term (≥37 weeks: 72.2%), and it was comparable in the two groups. Birthweight categories also showed no significant difference between groups (p = 0.222), with most infants weighing between 2.5 and 3.5 kg. Mode of delivery was predominantly via lower segment cesarean section (LSCS: 61.1%), and APGAR scores at five minutes were ≥7 in 95% of neonates, indicating overall good neonatal condition across both groups.

Counseling was conducted on day one in 58.3% of cases, between days two and five in 34.4%, and after day six in 7.2%, with uniform distribution across both groups (p = 0.281). The father's age, education, and religion were comparable between the two groups. Diagnostic categories included neonatal jaundice (NNJ: 27.2%), respiratory distress syndrome (RDS: 10%), late-onset neonatal sepsis (LONS: 8.3%), transient tachypnea of the newborn (TTNB: 7.8%), and others (46.1%). The distribution of diagnoses was balanced across groups (p = 0.484), further supporting comparability.

## Discussion

This study provides early evidence that integrating an AI chatbot into NICU counseling can substantially enhance the parental experience. To our knowledge, this is among the first evaluations of AI-assisted counseling in neonatology. Our findings align with broader international research on AI in healthcare communication and have important implications.

Significantly higher satisfaction scores in the AI-integrated group suggest that parents valued the clarity and depth of information provided. Traditional NICU counseling often compresses complex topics into brief conversations. The AI extension enabled clinicians to provide detailed, simplified responses to specific parental queries. Parents are left with a clearer understanding and fewer unanswered concerns, a known contributor to reduced anxiety and greater satisfaction [12]. This mirrors Kadivar et al. (2017), who reported increased maternal satisfaction following an internet-based NICU education program [12]. Similarly, under medical supervision, our chatbot served as an on-demand knowledge tool during high-stress, time-limited counseling [20].

This study preserved the clinician-parent bond. Instead of letting parents interact directly with AI, which may reduce trust, the clinician-curated AI-derived responses. This approach enhanced human interaction rather than replacing it, addressing concerns that AI could dehumanize care [16]. The hybrid model of empathy plus AI-backed knowledge echoes the broader "AI with human" model, which is gaining traction in healthcare [21]. Dwyer et al. (2023) demonstrated that an AI chatbot reassured postoperative orthopedic patients and reduced unnecessary care-seeking [22]. While our context differs, both studies highlight that timely, informative AI responses can alleviate stress. Koh et al. (2024) also found ChatGPT useful for counseling HIV patients, improving understanding and adherence [14]. Importantly, benefits were consistent across demographic groups. Even less-educated parents expressed high satisfaction, likely due to the clinician's role in filtering AI responses into understandable language. This is crucial, as health communication must be accessible to all. In our study, clinicians used the local language (Hindi) and analogies to simplify difficult concepts, a known strategy to improve caregiver comprehension [7].

AI responses, while generally accurate, required clinician review to eliminate jargon or inaccuracies. For example, a complex explanation of periventricular leukomalacia was distilled by the neonatologist into a simple summary of risks and follow-up plans. This aligns with Gray et al. (2024), who found that while ChatGPT-generated prenatal dialogues were helpful, expert oversight was essential [23].

We also considered how transparency around AI use affects trust. Parents were not explicitly told about the AI's involvement; information was presented as clinician-curated. This may have helped bypass trust barriers. A survey found that satisfaction with AI responses decreased slightly when participants knew the answers were generated by AI [24]. Our approach helped bridge this trust gap. As AI becomes more accepted, future implementations may allow for more transparency without compromising satisfaction. Notably, a NICU nurse interview study from Turkey voiced strong concerns about AI reliability [17], emphasizing the need for clear protocols and safeguards [25,26].

Globally, our study contributes to the recognition that generative AI can enhance patient-centered care. From oncology to primary care, AI chatbots are being piloted for FAQs, appointment reminders, and behavioral therapy [27]. The growing consensus is that AI improves consistency and access to information but must be guided by ethical standards and user needs [15]. In NICUs, AI could help ensure that parents, regardless of time or staff availability, receive accurate, timely answers. One can envision future bedside AI tablets allowing parents to ask questions anytime, with responses also visible to staff for follow-up. This could address the frequent information gaps parents experience outside of physician rounds. However, such systems must ensure data confidentiality, mitigate biased responses, and clarify professional responsibility. In our study, the clinician remained accountable for the information. Broader adoption would require certified, validated AI models, similar to how medical devices are regulated.

A key insight was that AI may enhance perceived empathy [28]. Our satisfaction scores were higher in the AI group, even for emotional support. This may reflect that AI helped clinicians focus more on emotional tone and less on information recall. Knowing they had AI support, clinicians may have felt more at ease and better equipped, enabling more empathetic, unhurried communication. This aligns with the literature suggesting that AI could ease clinician burnout in communication-heavy roles, allowing humans to manage emotional nuances while AI handles cognitive load.

While many studies explore AI in neonatal diagnostics or prognosis [15], few focus on direct parent interaction and AI's role in family-centered neonatal care. We propose future studies to explore cultural differences in AI reception, the inclusion of mothers, and additional outcomes such as reduced anxiety or improved care engagement. Trials should assess whether satisfaction translates into tangible benefits for both families and infant outcomes.

In summary, our findings support the integration of AI into NICU counseling as a means to strengthen, not replace, human connection. AI helped clinicians deliver more informative, empathetic, and individualized support. International literature supports this synergy model [18]. To scale up responsibly, stakeholders must address trust, training, and ethical safeguards. With thoughtful implementation, AI counseling tools could become a cornerstone of equitable, family-centered NICU care, ensuring every parent receives clear, compassionate answers when they need them most.

This study has several limitations. It was conducted in a single NICU in India, limiting generalizability across diverse settings. Only fathers were included, omitting maternal perspectives. Satisfaction was measured immediately post-counseling, without assessing long-term impact or information retention. The intervention was unblinded, introducing potential bias from both clinicians and participants. We used ChatGPT (GPT 4o), and results may differ with newer or specialized models, and real-time internet and manual vernacular translation may limit scalability. The accuracy of AI responses was not systematically evaluated, relying on clinician judgment. Satisfaction, a subjective outcome, was the sole metric; objective knowledge gain, anxiety reduction, or long-term outcomes were not assessed. Additionally, participant attitudes toward AI were not measured, and the effect of disclosing AI use remains unexplored.

## Conclusions

Despite its limitations, this study highlights the potential of generative AI-integrated counseling to enhance the parental experience in NICUs. The significant improvement in paternal satisfaction suggests that AI can serve as a valuable adjunct to traditional communication, enriching information delivery while preserving human empathy. Our findings support further exploration and refinement of this hybrid model across diverse settings and populations. As AI tools evolve, their responsible, context-sensitive integration into neonatal care could meaningfully contribute to family-centered practices, ensuring that parents feel better informed, supported, and engaged during one of the most challenging periods of their lives.

## Appendices

Annexure 1

**Table 3:** Questionnaire on parental satisfaction score

Q. No.	Question	1	2	3	4	5
1	Were you satisfied with the neonatologist's counseling regarding your newborn's NICU admission?	Not satisfied at all	A little satisfied	Satisfied to some extent	Partially satisfied	Completely satisfied
2	How well did you understand the information provided during the counseling?	Did not understand at all	Did not understand much	Understood to a limited extent	Partially understood	Completely understood
3	Were your concerns adequately addressed during the counseling session?	Were not addressed at all	Were not addressed as much as wanted	Addressed to a limited extent	Partially addressed	Concerns were completely addressed
4	How confident do you feel about your newborn's treatment and well-being after the counseling session?	Do not feel confident at all	Feel a little confident	Confident to some extent	Partially confident	Completely confident
5	Could you openly discuss your doubts and queries with the neonatologist during the counseling?	Could not discuss at all	Only discussed a little	Discussed to some extent	Partially discussed	All doubts and queries were discussed
6	How satisfied are you with the clarity of information provided during counseling regarding your newborn's health?	Not satisfied at all	A little satisfied	Satisfied to some extent	Partially satisfied	Completely satisfied
7	Did the neonatologist explain the treatment plan for your newborn in a way that was easy for you to grasp?	Did not understand at all	Understood a little	Understood to some extent	Partially understood	Completely understood
8	How well were you involved in the decision-making process for your newborn's care?	Was not involved at all	Involved a little	Involved to some extent	Partially involved	Completely involved
9	Were you provided with resources or additional support to help you better understand and manage your newborn's health condition?	Were not provided at all	Few resources were provided	Provided to some extent	Resources were partially provided	All resources were provided
10	How would you rate the overall satisfaction with the counseling and support for your family during this challenging time?	Not satisfied at all	A little satisfied	Satisfied to some extent	Partially satisfied	Completely satisfied
